# The challenges of nutrition policymaking

**DOI:** 10.1186/s12937-015-0001-8

**Published:** 2015-02-07

**Authors:** Joanne L Slavin

**Affiliations:** Department of Food Science and Nutrition, University of Minnesota, 1334 Eckles Avenue, St. Paul, Minneapolis, MN 55108 USA

**Keywords:** Dietary guidance, Nutrition policy, Evidence based review, Sodium, Added sugars

## Abstract

In my over three decades of work in the field of food and nutrition, I have participated in many efforts that seek new policy initiatives in the hopes that these programs can curb rates of obesity and chronic disease and help consumers make healthier dietary choices. Because of the profound effect that many of these policies have on consumers, the food environment, federal nutrition assistance programs and subsequent policy and regulatory recommendations, it is imperative that only the strongest, best available evidence is used to set policy. This review evaluates methods by which current nutrition policies use scientific research as well as provides recommendations for how best to ensure future nutrition policies are truly science-based and likely to have a meaningful impact on public health. Specifically, this review will:

- Describe the current food and nutrition policy environment in the US
- Examine how science is used in federal food and nutrition policymaking efforts, using the Dietary Guidelines for Americans (DGA) as an example
- Describe strong versus weak science as well as what types of studies are most appropriate for use in policymaking
- Discuss the potential effects and consequences of making policy recommendations in the absence of scientific consensus or agreement
- Make recommendations to support the present and ongoing development of science-based policy likely to positively impact public health

## Introduction

The US food and nutrition policy and regulatory environment is highly active. The current administration, federal agencies and regulators are increasingly looking to policy and systems-change interventions to improve public health in America. For example, within the last five years, federal and state/local governments have instituted significant changes to the school food environment [1], proposed state and local initiatives to tax and/or ban certain foods and beverages [2], and published proposed rules to significantly change nutrition labeling regulations [3]. Additionally, the 2015 Dietary Guidelines Advisory Committee (DGAC) [4] is presently meeting and will issue the 2015 DGAC report in the coming year.

The process by which federal agencies and policymakers consult scientific research in developing proposed regulations and policies varies, and greatly impacts the nature of the ultimate recommendations. An investigation into this process would yield important understanding about how science is used to set policy and what impact this process is likely to have on consumers.

## Review

### How science is used in policymaking

Science is used by all agencies to set nutrition policy. Yet, guidelines for how to identify, evaluate, and translate scientific research into policy recommendations vary among agencies. Policymakers generally rely on published research and consensus reports by scientific authorities and government bodies; however the manner in which research findings and report conclusions are interpreted and applied can differ from one initiative to the next. Government agencies have outlined their approach for evaluation of scientific studies to be used in decision-making. For example, NIH uses the AHRQ system [5] and FDA has an accepted system of systematic review for health claims [6]. Because there is not a universally accepted evidence-grading scheme, conclusions are based on research studies with varying degrees of methodological strength and applicability. The fact that nutrition research produces constantly evolving scientific findings further complicates the development of objective, evidence-based policy recommendations.

One example of a US scientific authority with significant influence is the Institute of Medicine (IOM). The IOM is one of the premier authoritative bodies that conducts health-related research and promulgates health and nutrition recommendations for policymaking purposes. IOM reports are frequently commissioned by government agencies for topics where policy and/or regulatory interest exists but research gaps remain. Some recent examples include sodium [7] and front-of-package labeling [8]. Once IOM recommendations are published, they are often used as scientific basis for proposed regulations and nutrition guidance. IOM recommendations aim to reflect our most current scientific understanding and usually precede the actual setting of policy to ensure any action is evidence-based. However, the IOM is challenged to keep pace with advances in our understanding of nutrition.

For example, the IOM completes the Dietary Reference Intake (DRI) reports, which are considered the most reliable sources of nutrient recommendations – they inform the very basis of our current nutrition understanding. The DRIs are summarized in the 2006 volume [9] and are an update to the Dietary Recommended Allowances (RDA) that have been published since 1941. While DRI reports for certain nutrients have been updated recently (vitamin D and calcium were updated in 2011), other DRI reports have not been updated since 1997–1998. This means that the body of research that has been completed for a number of nutrients within the last 15-plus years is not accounted for in our current IOM DRI report conclusions.

Researchers and policymakers also rely heavily on the National Health and Nutrition Examination Survey (NHANES), an ongoing group of studies designed to assess the health and nutritional status of adults and children in the United States. These studies are based on self-reporting; they consist of 24-hour dietary recalls completed through individual surveys. NHANES also collects biological data and anthropometrical data with mobile units. NHANES information is a valuable resource on changes in nutrient intake and health status of a cross-sectional group of US consumers.

Critics suggest the data are flawed because of biases that accompany self-reporting measures [10]. As one might expect, survey respondents have a tendency to under-report their caloric intake or over-report the amount of more nutritious foods they consume and under-report the amount of less nutritious foods they consume. Archer et al. [11] reported that 67% of women and 59% of men who participated in NHANES provided caloric intake responses that were not physiologically plausible. They calculated physiologically credible energy intake values as the ratio of reported energy intake to estimated basal metabolic rate and subtracted estimated total energy expenditure to create disparity values. The greatest mean disparity values were – 716 kcal/day and −856 kcal/day for obese men and women, respectively. The limitations of our nutritional data are generally not acknowledged in scientific reports or consensus statements. And yet, NHANES is cited by virtually every government agency involved in health and nutrition as an accurate representation of Americans’ eating habits.

These examples raise important questions about the data that US nutrition policymakers have available to them. How confident can we be that federal dietary guidance is evidence-based when our foundational measures are outdated and significantly limited? What controls can be put in place to ensure that policies and regulations are likely to have demonstrated, positive public health impact?

### The dietary guidelines advisory committee

Another highly influential scientific authority is the Dietary Guidelines Advisory Committee (DGAC), the appointed review committee responsible for formulating and publishing (in the form of a comprehensive report) an evidence-based review that provides scientific support for the Dietary Guidelines for Americans (DGA) policy document. The DGA are statutorily mandated (Section 301 of Public Law 101–445 (7 U.S.C. 5341, the National Nutrition Monitoring and Related Research Act of 1990, Title III)) and are a collaborative effort between the Department of Health and Human Services (HHS) and Department of Agriculture (USDA); the DGA have been published every five years since 1980. The DGA aim to provide “sound advice for making food and physical activity choices that promote good health, a healthy weight, and help prevent disease for Americans ages 2 years and over, including Americans at increased risk of chronic disease” [12]. DGA recommendations serve as the cornerstone for all Federal nutrition education and program activities, including but not limited to nutrition labeling campaigns by the Food and Drug Administration (FDA) and USDA Food Safety and Inspection Service (FSIS), the Office of Disease Prevention and Health Promotion (ODPHP) *Healthy People* objectives, and USDA Food and Nutrition Service nutrition assistance programs including the Supplemental Nutrition Assistance Program (SNAP) and the National School Lunch Program (NSLP). As a result, DGA reach and impact are extensive.

The 2015 DGA process is underway, with the current DGAC holding meetings to share their evidence review process and findings with the general public. According to the 2015 DGAC charter, the Committee’s official responsibilities are to “examine the current *Dietary Guidelines for Americans*, take into consideration new scientific evidence and current resource documents, and then develop a report to be submitted to the Secretaries that outlines its science-based recommendations and rationale which will serve as a basis for developing the eighth edition of *Dietary Guidelines for Americans*” [12].

The DGAC is governed by Federal Advisory Committee Act (FACA) guidelines and an official charter and charge [13]. While the freedom exists to explore food and nutrition topics that the DGAC deems important and scientifically relevant, the charge explicitly states that “DGAC responsibilities include providing authorship for this report; however, responsibilities do not include translating the recommendations into policy or into communication and outreach documents or programs” [13]. In other words, DGAC recommendations should be scientific in nature and not indicative of policy direction.

### The DGAC evidence review process

The DGAC process to identify, review, and evaluate available nutrition research for a variety of topics is complex and time-intensive. Typically, DGAC members are divided into subcommittees to address specific research areas based on topic importance and DGAC member expertise. In 2010, the DGAC consisted of thirteen scientists with expertise in nutrition, physical activity, food behavior and nutrition through the lifecycle. There were eight subcommittees focusing on the following dietary issues: 1) alcohol; 2) carbohydrate; 3) energy balance and weight maintenance; 4) fatty acids and cholesterol; 5) food safety and technology; 6) nutrient adequacy; 7) protein; and, 8) sodium, potassium and water. As a member of the 2010 DGAC, the author of this paper served as chair of the carbohydrate and protein subcommittees and also as a member of the energy balance and the nutrient adequacy subcommittees.

The 2015 DGAC is organized somewhat differently, with fourteen scientists serving on five subcommittees: 1) Food and Nutrient Intakes, and Health: 2) Current Status and Trends; Dietary Patterns, Foods and Nutrients, and Health Outcomes; 3) Diet and Physical Activity Behavior Change; 4) Food and Physical Activity Environments; and 5) Food Sustainability and Safety. There are separate working groups for sodium, added sugar, saturated fat and physical activity. The 2015 Committee is also using expert consultants to inform its evidence reviews.

One of the first steps in the DGAC evidence review process is to develop research questions regarding the relationship between diet and health outcomes, including disease risk or health benefits (e.g., what is the relationship between dietary fiber intake and specific health outcomes). These questions should reflect the research gaps identified by the previous DGAC, as well as areas of nutrition where there is new, influential evidence since the previous edition of the DGA. Once the research questions have been agreed upon, the DGAC, in concert with USDA Nutrition Evidence Library (NEL) staff, gathers the relevant available studies.

The research studies are then closely examined and evaluated based on strength of study design as well as relevance of outcomes. In past years, the DGAC used the NEL evidence-based review process [14], a strict hierarchy of evidence and rigorous grading process. For each question addressed in the 2010 evidence-based report, the DGAC developed precise search criteria, inclusion and exclusion criteria for all of the studies, including the range of dates searched, and made this information available on the USDA DGA portal [14]. Such detailed process and transparency in the NEL evidence-based approach minimizes bias and therefore adds credibility to the findings. However, the scientific review method ultimately used by the DGAC is at the Committee’s discretion – for example, at the time of this paper’s completion, the 2015 DGAC has decided to use the NEL process to answer some research questions, but not others. This permitted subjectivity and variability increases the potential for less rigorous studies to be used to inform DGAC recommendations.

Once the DGAC has determined which studies to examine for each research question, evidence conclusion statements are written. Within the NEL system, the conclusions drawn can be deemed as strong, moderate, limited, or lacking data to support them. There may also be strong evidence of no relationship. For example, strong evidence was found of no relationship between glycemic index and disease outcomes in the 2010 DGAC review [15]. Agreeing on the strength of the relationship is always difficult, as for each question, different types of studies with a variety of outcomes have been published. A closer examination of study methodology will help further illustrate this point.

The DGAC process is transparent and open to input from scientists and consumers. The 2015 DGAC will hold 7 public meetings with public comments accepted throughout the process. Although the final DGAC report is not released, the committee regularly updates their progress on reviewing scientific questions at the public meetings.

### Research methodology: what makes a strong vs. weak study

The evidence-based medicine (EBM) hierarchy ranks research design in the following order of strength (from highest to lowest): systematic reviews of randomized-controlled trials (RCT), RCT, prospective cohort studies, case control studies, cross-sectional studies, case series/case reports and editorials/expert opinions. RCT are the strongest study designs for determining cause and effect between a dietary exposure and a health outcome [16]. Following RCT are prospective cohort studies, where a group or cohort of subjects is studied over time. Food frequency instruments are often used to collect dietary information before any diagnosis of disease, making these studies more reliable than cross-sectional studies where diet and outcome measures are assessed simultaneously. Historically, in the case of DGAC reviews, no case–control studies, animal research, or *in vitro* studies have been considered due to their relative weakness and because their findings cannot prove cause and effect in humans. Typically cross-sectional studies are only included in DGAC reviews if no stronger prospective studies are available.

Following this reasoning, food and nutrition policies would be best served if only the strongest types of evidence – perhaps RCT alone – informed their development. However, this is an unrealistic ideal as not all diet and health outcome relationships can be practically or ethically evaluated using RCT. For example, it is difficult to carry out blind food treatments in dietary studies (subjects know they are consuming an apple versus apple juice). However, such trials can work with nutrients, as nutrients can be added to food or drinks without the knowledge of the participants or investigators (the double-blind mechanism).

Further, all RCT data are not created equal. RCT generally use biomarkers as outcome measures rather than disease incidence due to the length of time it takes healthy people to manifest disease symptoms. Biomarker data can be extrapolated to infer relationships regarding population health without adequately accounting for weaknesses in the relationship between the biomarker and the disease state. Ultimately, this can result in a strong study methodology being misapplied and used to make assumptions that are not actually supported by the research. For example, RCT are clear that sodium intake or excretion is directly related to blood pressure, yet prospective cohort studies show that too low sodium intakes actually increase risk of cardiovascular disease (CVD). Thus, at low levels of sodium consumption, blood pressure does not account for all of the CVD risks. Biomarker data fail to tell the complete story.

In reality, many dietary recommendations are supported by evidence primarily from observational data, particularly those from prospective, cohort studies. Nutrition scientists and policymakers often underappreciate limitations of such data. Some of the limitations of observational evidence for diet-disease relationships include imprecise exposure measures, collinearity among dietary exposures, displacement/substitution effects, healthy/unhealthy consumer bias, and residual confounding. Maki et al. [16] recommend greater caution in making dietary recommendations for which RCT evidence of clinical event reduction after dietary intervention is not available.

For these reasons and because nutrition science is complex and changeable, it is critical that study methodology is carefully considered and applied to our interpretation of nutrition science. Ideally, observational data would be validated by stronger research methods before being used to inform policy. While observational research may be valuable to our understanding of nutrition and health, its limitations must be acknowledged. Consider the 2015 DGAC investigation into sustainable dietary patterns. This field of research is arguably in its infancy – in fact, there is no scientific consensus for even a definition of sustainability [17]. Any sustainability-related recommendations in the 2015 DGAC report should be preliminary at best, recognizing the need for additional, rigorous research to validate initial findings. Without these underlying studies in place, it would be premature for HHS and USDA to use sustainability recommendations to inform nutrition guidance in the 2015 DGA policy document.

### Consequences of non-evidence-based policy

We don’t have to travel very far back in time to witness examples of dietary guidance recommendations that were made prematurely and are now challenged as more research is introduced. Our understanding of fats has evolved considerably, with dietary recommendations now emphasizing healthy consumption of monounsaturated and polyunsaturated fats, proving that healthier dietary patterns include, rather than exclude, foods higher in fat content.

More recently, it could be argued that the 2010 DGA sodium intake recommendation was made in the absence of scientific consensus. The policy document recommends that individuals over 51 years old, African Americans or those with hypertension, diabetes, or chronic kidney disease reduce their daily sodium intake to 1,500 milligrams. This applies to about half the US population, including children and the majority of adults.

Since then, the IOM published its *Sodium Intake in Populations: Assessment of Evidence* report. Findings stated that recent studies “support current efforts to reduce excessive sodium intake in order to lower risk of heart disease and stroke. However, the evidence on health outcomes is not consistent with efforts that encourage lowering of dietary sodium in the general population to 1,500 mg/day. Further research may shed more light on the association between lower—1,500 to 2,300 mg—levels of sodium and health outcomes [7].

The 2010 DGA recommendations are now inconsistent with our most recent scientific understanding of sodium and health. As noted, this conflict could have been avoided if the DGA policy document had withheld such extreme guidance until more rigorous studies were fielded, reviewed, and published. Recent papers in the New England Journal of Medicine cast further doubt on our low sodium recommendations for the general public [18].

The sodium example is important because of the aforementioned impact of DGA recommendations on other food and nutrition policies. The Final Rule for the Nutrition Standards in the National School Lunch and School Breakfast Programs [1] states that schools must “reduce the sodium content of meals gradually over a 10-year period through two intermediate sodium targets of two and four years post implementation”. Now that schools have begun to implement the new regulations, these severe sodium reductions are proving difficult, costly, and may reduce student participation rates [19]. These consequences are especially concerning considering the underlying recommendation may not accurately reflect the current evidence base.

Inaccurate and conflicting dietary guidance messages are also detrimental to consumers’ understanding of nutrition and their ability to build healthy diets. At a time when consumers are already subjected to an overabundance of nutrition and health information, government agencies should be held accountable for developing policies and regulations that are rooted in strong science, and are realistic and achievable for the majority of the population. In the case of sodium, not only is there insufficient evidence to link highly restrictive sodium intakes to improved health outcomes, but encouraging the general public to reduce intakes from the estimated current average of 3,400 mg/day to 1,500 mg/day is self-defeating and unachievable [20].

Another example can be seen in the use of the 2010 DGAC review to support the FDA proposal to mandate added sugars labeling on the Nutrition Facts panel [3]. Added sugars have become the current nutrition “watch out”, believed by some to uniquely contribute to obesity and other adverse health outcomes. However, the majority of scientific evidence shows that all sugars (added or intrinsic) provide 4 kcalories/gram just like any other digestible carbohydrate and are no more likely to cause weight gain or negative health outcomes than other calorie sources [21]. In fact, even the proposed rule acknowledges this fact:

“U.S. consensus reports have determined that inadequate evidence exists to support the direct contribution of added sugars to obesity or heart disease. Specifically, although it is recognized that sugar-sweetened beverages increase adiposity (body fat) in children (Ref. 30), neither the 2010 DGA nor the IOM macronutrient report concluded that added sugars consumption from all dietary sources, in itself, increases obesity. In fact, the 2010 DGA states that added sugars do not contribute to weight gain more than any other source of calories…” [3].

FDA states that the basis for this proposed labeling requirement is the 2010 DGA recommendation to reduce intakes of added sugars to assist consumers in maintaining healthy dietary practices. The DGA rationale is that lower intakes of added sugars will result in decreased calorie intakes and increased nutrient density of individual diets, not reduced risk of adverse health outcomes. Specifically, the 2010 DGAC energy balance subcommittee investigated sugar-sweetened beverage intakes and found that “strong evidence shows that children who consume more sugar-sweetened beverages have greater adiposity (body fat) compared to those with a lower intake” [15]. However, a closer look at the evidence review shows that only 12 of the 19 studies (which included cross-sectional studies) found a positive association between sugar-sweetened beverage intakes and adiposity in all or a subsample of population studies. It is difficult to see how the subcommittee concluded this to be “strong” evidence.

Furthermore, it is unclear why FDA proposed mandatory added sugars labeling in the absence of consumer research to demonstrate whether the change will in fact influence consumer understanding and purchasing behavior. The proposed rules even preceded the agency’s own study. Existing consumer research suggests that consumers already find aspects of the current nutrition label confusing [22]. In addition, public misunderstanding about added sugars abounds. Some consumers believe added sugars do cause unique adverse health outcomes compared to other sugars and even contain more calories that intrinsic sugars [22]. Even if the intention behind the proposed rule is to steer consumers away from purchasing non-nutrient dense foods and beverages that contain added sugars, current available research suggests they will do this for the wrong reasons. This proposal stands to perpetuate misleading beliefs about nutrition and lead to more consumer confusion.

It is extremely difficult to reverse or change public policy, once enacted, without causing consumer confusion. There are few mechanisms available to regulators and policymakers to make adjustments that reflect new science and understanding. Furthermore, nutrition policy recommendations, once adopted, appear frequently in the media and online. Reversing consumer misunderstanding about nutrition is an incredibly difficult task; providing the public with accurate, realistic and achievable information first would go a long way in improving our understanding of nutrition and health, and ultimately contributing to improved public health outcomes.

## Conclusions

It is imperative that food and nutrition policies reflect, and do not get ahead of the strongest available scientific evidence. It is unlikely we will ever have RCT data available to answer most nutrition questions, but we should rely on our strongest designs, including prospective cohort studies. We should not accept cross-sectional studies as influential drivers of policy development. We must demand stronger scientific standards from our appointed committee members who serve on advisory IOM and DGAC panels.

A transparent system that grades evidence quality would help achieve consistency in science interpretation and use across nutrition policies and regulations. Grading schemes should be vetted and discussed by experts across the wide expertise needed in dietary guidance, including nutritionists, dietitians, food scientists, physicians, applied economists, and food processors so that findings and recommendations could be supported across a wide array of credible groups. This would also help ensure that the dietary guidance messages consumers are receiving are factual and consistent.

When policy recommendations are developed by committees, such as the DGAC, those committees should be comprised of a balanced and well-rounded set of perspectives and expertise. Ideally a scientific nutrition committee would not only include experts in nutrition, biochemistry, physiology, epidemiology and statistics, but also food science, food production and processing, food policy and behavior. This combination of skills would ensure that the ultimate recommendations adequately reflect our entire food system and food environment.

Scientists who understand how we “learn” about nutrition must be included, even if they have worked on research supported by commodity groups or food companies. The IOM process considers bias of individual committee members and whether they have taken such strong public stands on issues that it is not possible for them to move to another position based on the deliberations of the committee. Any linkages to the food industry are criticized, yet there seems to be little concern about committee members who are closely linked to professional groups, such as American Heart Association or other advocacy groups. Improvements to our food system and public health can only be realized if we work together, respecting the strengths of all parties. Nutrition advice that is produced in such a collaborative system will more likely be translatable and realistic for the general public.

Policies should reflect what is practical and likely to have the most impact on the general population. Simple, flexible and straightforward messages that are rooted in the best available evidence are likely to be most effective. For example, the majority of Americans are unlikely to be interested in or able to prioritize building sustainable diets, shop at farmers markets, or avoid processed foods, which provide nutrition and convenience for individuals with less access to full-service grocery stores and fresh produce.

I would finally suggest that the US government consider elongating the DGA publication schedule. The DRI reports and nutrition labeling regulations are not updated every 5 years; instead they are reexamined when there is a sufficient level of new research to warrant a change. Without new science to review, the DGAC may choose to focus on fads and trends instead of updating the scientific data for the core areas of dietary guidance. As every DGAC wants to be bold and set new direction, nutrition science would support that first we must do no harm with our dietary guidance. Moderation and variety must be kept front and center, as well as an appreciation that a teenage active boy may need 2 or 3 times more calories than an elderly man or young child. A suggestion that all Americans should reduce sodium intakes is not sound and is potentially dangerous. Targeting certain foods and beverages, including chocolate milk, processed meats, added sugars, and even the noble potato as villains in the nutrition wars is not a science-based strategy and may need to be countered on the political front if appointed scientific review committees continue to take this approach.

As described by Schneeman [6], science is necessary for developing effective food regulation and policy, but it is not sufficient. The interface between nutrition and public health must include food science and agriculture. Food technology can help all consumers, including those of lower socioeconomic status, have access to safe, nutritious foods that science has found to be linked to improved health outcomes.

## Abbreviations

HHS: Department of Health and Human Services
USDA: Department of Agriculture
DGAC: Dietary Guidelines Advisory Committee
DGA: Dietary Guidelines for Americans
DRI: Dietary reference intakes
EBM: Evidence-based medicine
FACA: Federal Advisory Committee Act
FDA: Food and Drug Administration
IOM: Institute of Medicine
LDL: Low-density lipoprotein
NHANES: National Health and Nutrition Examination Survey
NSLP: National School Lunch Program
ODPHP: Office of Disease Prevention and Health Promotion
RCT: Randomized-controlled trials
SNAP: Supplemental Nutrition Assistance Program
FSIS: USDA Food Safety and Inspection Service
NEL: USDA Nutrition Evidence Library
